# Knowledge and Attitudes Toward Human Papillomavirus Vaccination Among Latina Mothers of South American and Caribbean Descent in the Eastern US

**DOI:** 10.1089/heq.2018.0058

**Published:** 2019-05-30

**Authors:** Rula Btoush, Diane R. Brown, Jennifer Tsui, Lindsey Toler, Jennifer Bucalo

**Affiliations:** ^1^ School of Nursing, Rutgers University, Newark, New Jersey.; ^2^ School of Public Health, Rutgers University, Newark, New Jersey.; ^3^ Cancer Institute of New Jersey, Rutgers University, New Brunswick, New Jersey.; ^4^ School of Public Health, Rutgers University, Piscataway, New Jersey.; ^5^ School of Graduate Studies, Rutgers University, Newark, New Jersey.

**Keywords:** papillomavirus vaccines, health behavior, adolescent health, cervical cancer

## Abstract

**Purpose:** The purpose of this study was to examine knowledge, attitudes, barriers, and facilitators for human papillomavirus (HPV) vaccination among Latina mothers of HPV vaccine-eligible children in low-income urban areas, as well as useful strategies to improve HPV vaccination.

**Methods:** The study included 132 Latina mothers of HPV vaccine-eligible children, interviewed in 14 focus groups. Using semi-structured discussions, mothers were asked about their knowledge about HPV infection and vaccine, views toward HPV vaccination, barriers for HPV vaccine initiation as well as completion, and opinions on strategies to improve HPV vaccination.

**Results:** Only 55% of mothers reported having ever heard of the HPV vaccine, 27% of mothers indicated initiating the HPV vaccine, and 14% indicated completing the multi-dose series. Mothers generally lacked knowledge about HPV infection and vaccination, with varying degrees by Latino descent. Health care provider (HCP) recommendation was the strongest barrier/facilitator for HPV vaccination. Useful strategies to improve HPV vaccine initiation and completion that the mothers suggested included strong recommendation from HCPs and addressing side effects and safety concerns. Other useful strategies included community and school-based approaches and the use of text messaging and smartphone technology to educate mothers and send vaccine reminders.

**Conclusion:** The findings provide insight for the development of interventions targeting low-income Latina mothers and the need to improve HCP communication on HPV vaccination.

## Background

There are pervasive disparities in national morbidity and mortality rates of human papillomavirus (HPV)-related cancers for Latino individuals.^1^ In New Jersey, cervical cancer morbidity is significantly higher in the Greater Newark area as well as among Latina women.^2^ In 2015, the HPV vaccine uptake rates among female and male adolescents nationally were 63% and 50% for initiation, and 42% and 28% for completion of the three-dose series.^3^ These rates are dramatically lower than *Healthy People 2020's* goal of achieving 80% completion rate.^4^ Further, even though national data show slightly higher HPV vaccination among Latino and low-income groups,^3,5–7^ local-level data and several community-based studies have demonstrated a significant and continuing trend of lower HPV vaccination among Latino adolescents,^8–14^ as well as in low-income and urban areas.^11,15–17^ Research in the Greater Newark area revealed that only 21% of Latino adolescents (10–20 years old) have initiated the HPV vaccine,^18^ and 8% have completed the three-dose series.^19^

Mothers' knowledge about the HPV vaccine has been found as a strong predictor and important facilitator for adolescent uptake of the HPV vaccine.^20–23^ Studies have demonstrated that even though Latina mothers have low levels of knowledge about the HPV infection and vaccination,^24–27^ they are accepting of and have positive attitudes toward HPV vaccination.^23–25^ Barriers for the HPV vaccine among Latina mothers include concerns about cost of the vaccine,^28^ safety and potential side effects,^26^ and the belief that their children were not sexually active and therefore not susceptible to HPV infection.^21^

Only a few studies have recently used qualitative methodology to explore knowledge and attitudes of Latina mothers around HPV vaccination.^29–31^ Morales-Campos et al.^29^ found gaps in knowledge about HPV vaccination among Latina mothers, fears and concerns about the vaccine, and Latina mothers' desire to protect their children through vaccination. Aragones et al.^30^ found low levels of knowledge around HPV infection and vaccination and lack of provider recommendation as the main barrier to vaccination. Similarly, Albright et al.^31^ reported that the most commonly reported barrier among Spanish-speaking parents is when providers do not encourage the initiation of the HPV vaccine or do not explain the necessity of completing the series. These studies, however, describe the knowledge and attitudes of Latina mothers as one single group without investigating potential difference across different descent groups.

In conclusion, although a few studies have examined knowledge and attitudes toward HPV vaccination among Latina mothers, literature is still lacking in providing an in-depth investigation of the barriers and facilitators, beyond standard survey measures, for both HPV vaccine initiation and completion as well as for both female and male adolescents. Also, very little is known about the differences in knowledge and attitudes among Latina mothers of different descents. Lastly, studies have not investigated mothers' perceptions of useful strategies to improve HPV vaccine uptake among the Latino adolescent population in low-income areas. Therefore, the purpose of this study is to examine knowledge and attitudes toward HPV vaccination among Latina mothers of South American and Caribbean descent in low-income urban areas of the Eastern United States. The study objectives are to:

(1) Assess knowledge, attitudes, and acceptability for HPV vaccination for girls and boys.
(2) Identify barriers and facilitators for HPV vaccination initiation and completion identified by the mothers.
(3) Examine cultural and other factors that are useful for the development of targeted interventions to promote HPV vaccination among this population.

## Methods

This is a qualitative study examining knowledge and attitudes toward HPV vaccination among Latina mothers. The study was approved by Rutgers Institutional Review Board. Study recruitment and data collection occurred in January–December 2015. Participants were recruited from a multi-site community health center and a clinic of an urban community hospital in the Newark/Elizabeth area in New Jersey, through direct invitation, advertisement posters, and word of mouth. To be eligible for the study, participants had to be older than 18 years, self-identify as Latina, and have at least one child eligible for the HPV vaccine (i.e., between the ages of 11 and 18 years). According the 2010 US census data,^32^ Latinos made up 58% of the population in the five zip code areas targeted in this study (27% Caribbean Latinos, 18% South American, and 11% Central American).

Fourteen focus groups of Latina mothers were conducted at the recruitment sites with 8–12 participants each, for a total of 132 women. The final sample size was based on achieving data saturation. Twelve of the focus groups were conducted in Spanish and two focus groups were conducted in English, based on the participant's individual choice. Each focus group took 1–2 hours and was audio-taped. Two Spanish-speaking facilitators, who were also fluent in English, conducted the focus groups. The facilitators received training on active interviewing, facilitating discussions, engaging participants, and research ethics. The principal investigator was present during all of the focus groups as an observer and to provide any needed support for the facilitators.

The focus group questions, presented in Table 1, were developed based on the Health Belief Model,^33^ as well as on other studies on HPV vaccination.^34–36^ Participants received light snacks and refreshments and a $20 gift card to a local store as an incentive. Because the focus group discussions revealed considerable lack of knowledge regarding HPV infection and vaccination, the focus group facilitators led a brief educational session at the end of the focus group to correct misconceptions and answer any questions. Written information materials about HPV infection and vaccination were also distributed to participants in Spanish and English.

**Table 1.** Focus Group Questions

PART 1: KNOWLEDGE OF HPV
1. Before we talk about the HPV vaccine, what do you know about HPV infection?
*Probe: How is it transmitted? Who is at risk for the infection? Etc.*
PART II: KNOWLEDGE OF HPV VACCINATION
1. What do you know about HPV vaccination?
*Probes: What do you know about the HPV vaccination guidelines? Who should get the vaccine? At what age? How many shots? Etc.*
2. How did you learn about the HPV vaccination?
3. Has the doctor or nurse talked to you about the HPV vaccine? How did they discuss it with you?
PART III: VIEWS TOWARD THE HPV VACCINE
1. How do you feel about the HPV vaccine overall?
2. Tell us about the decision-making process in vaccinating your children.
*Probe: Discuss the role of the child's father, family, community, religion, media,* etc.
3. How do you feel about vaccinating adolescent girls?
4. How do you feel about vaccinating adolescent boys?
5. What would discourage mothers from vaccinating their children against the HPV infection?
6. What would motivate mothers to vaccinate their children against the HPV infection?
7. How can we best reach out to Latina mothers to educate them about the HPV vaccine?
PART III: QUESTIONS BASED ON HPV VACCINATION STATUS
Questions for mothers who have not to initiate the HPV vaccine:
1. You are in this group because you have not started the HPV vaccine for your adolescent child(ren). Could you discuss your decision with us?
*Probe: What made you decide not to start the vaccine? Were there specific individuals/situations that influenced your decision?*
2. What information or factors influence your future decision making about the HPV vaccine?
Questions for mothers who have initiated but not competed the HPV vaccine:
1. You are in this group because you have started the HPV vaccine for your adolescent child(ren) but did not complete all three shots. Could you discuss your decision with us?
*Probe: What made you decide not to complete the vaccine? Were there specific individuals/situations that influenced your decision?*
2. Do you intend on completing the vaccination shots? Explain why.
3. What information or factors influence your future decision or ability to complete the vaccine?
Questions for mothers who have competed the HPV vaccine:
1. You are in this group because you have completed the HPV vaccine for your adolescent child(ren). Could you discuss your decision with us?
*Probe: What made you decide to complete the vaccine? Were there specific individuals/situations that influenced your decision?*
2. Are there information or factors that assisted you in completing the HPV vaccine?

HPV, human papillomavirus.

Before the start of the focus group, participants were asked to complete a form providing their demographic information and indicating whether they had ever heard of the HPV vaccine, whether their children had received the HPV vaccine, and whether their children had completed the three-dose series of the HPV vaccine. This study was conducted before the dosing schedule for HPV vaccination was changed in 2016.^37^ Data from Table 2 show the study sample characteristics. Approximately half of the participants were between 21 and 55 years of age, and most (85%) spoke Spanish. Overall, 64% had high school education or less and 82% reported annual income less than $14,000. One-third (34%) were covered by Medicare or Medicaid; and 52% had no insurance. As shown in Table 3, bivariate analysis, using the Chi-square test, was conducted to examine demographic predictors for ever hearing of the HPV vaccine and for having received HPV vaccination.

**Table 2.** Characteristics of the Study Sample

	*N*	%
Origin		
Caribbean	55	41.7
South American	22	16.6
Central American	55	41.7
Country of Origin		
Columbia	5	3.8
Cuba	7	5.3
Dominican republic	20	15.2
Ecuador	35	26.5
El Salvador	7	5.3
Guatemala	1	0.8
Honduras	3	2.3
Mexico	11	8.3
Peru	12	9.1
Uruguay	2	1.5
The United States–Puerto Rico	28	21.2
Venezuela	1	0.8
Language		
English	19	15.2
Spanish	112	84.8
Education		
High school or less	85	64.4
More than high school	47	35.6
Employment status		
Unemployed	93	70.5
Full time	20	15.2
Part time	19	14.4
Income (annual)		
<$14,000	108	81.8
$15,000−$29,000	13	9.8
>$30,000	11	8.4
Marital status		
Married/partnered	82	62.1
Single	26	19.7
Divorced/separated/widowed	24	18.2
Insurance status		
Private insurance	19	14.4
Public (Medicaid/Medicare)	45	34.1
Uninsured	68	51.5
Ever heard of HPV vaccine		
Yes	73	55.3
No	59	44.7
HPV vaccination status		
Not vaccinated	95	72.0
Initiated by not completed	17	12.9
Completed	19	14.4

Continuous variables	Range	Mean (SD)
Age	21–55	38.6 (7.1)
Years in the United States	1–44	14.0 (9.5)
No. of children	1–6	2.6 (1.2)
No. of daughters	0–4	1.7 (0.9)
No. of sons	0–5	1.6 (0.8)

**Table 3.** Bivariate Analysis of Ever Hearing About and Receiving the Human Papillomavirus Vaccine

	Ever heard of HPV vaccine, %	Received HPV vaccination, %
Mother's age		
<40 years old	50.0	30.6
40 years old or more	61.7	23.3
Hispanic origin		
Caribbean	61.8	30.9
Central/South American	50.6	24.7
Language		
English	90.0^***^	45.0^*^
Spanish	49.1	24.1
Education		
High school or less	51.2	29.3
More than high school	62.0	24.0
Employment		
Unemployed	52.7	22.6
Employed	61.5	38.5
Income (annual)		
$14,000 or less	49.1^**^	24.1
More than $14,000	83.3	41.7
Mother's marital status		
Married/partnered	56.1	29.3
Single/separated/divorced	54.0	24.0
Insurance status		
Insured	64.1^*^	39.1^**^
Uninsured	47.1	16.2
Ever heard of the HPV vaccine		
Yes	—	46.6^***^
No	—	3.4

This table includes row percentages (e.g., among English speakers, 90% ever heard of the HPV vaccine).

^*^ *p*≤0.05, ^**^p≤0.01, ^***^p≤0.001 using Chi-square test.

The focus group discussions were transcribed and translated to English, with embedded links to the original transcripts in Spanish, to capture the essence and meaning of the data. Data analysis consisted of thematic content and standard focus group analysis techniques.^38^ A grounded theory approach was used for coding the data, identifying emergent themes, comparing for similarities and differences, and developing conceptual descriptions of themes.^39^ Transcripts and meeting notes were reviewed multiple times to identify common concepts and ideas, and consequently derive study themes along with verbatim phrases. Using the process of open coding, significant statements and concepts were identified and grouped together to form themes. A coding scheme was used to identify similarities in responses, conduct group comparisons, and provide quotes supporting the identified concepts and themes. Lastly, the data analysis compared differences and similarities in knowledge and perceptions separately for South and Central American (SCA) mothers and for Caribbean Latina (CL) mothers. Compared with CL mothers (from the United States–Puerto Rico, Cuba, and Dominican mothers), SCA mothers are more likely to be recent immigrants and uninsured, and to be less familiar with the health care system and vaccination guidelines. Therefore, the data analysis identified themes for the entire sample, as well as themes that are different for CL mothers versus SCA mothers. Table 4 provides a summary of the study results.

**Table 4.** Summary of Study Results

Knowledge about the HPV infection
Knowledge:
• Overall limited knowledge and confusion about HPV infection, methods of transmission, and its consequences.
• Most recognized the link between HPV infection and cervical cancer, but not other HPV-related cancers.
• Some confusion between HPV and HIV.
• Uncertainty about the risk factors for the infection and whether it affects women, men, or both genders.
• Misinformation about HPV transmission methods: by contact/touching, through childbirth, when exchanging underwear, by blood transfusion, by sharing needles, and through public places.
Varying levels of knowledge:
• CL mothers were more knowledgeable about the HPV infection and its consequences.
• SCA mothers lacked knowledge and had more misinformation about the HPV infection.
Sources of information:
• The most common source of information is the child's doctor.
• Other sources: internet, TV commercials, and through knowing others who were infected with HPV.
Knowledge about the HPV vaccine
Knowledge:
• Fifty-five percent have ever heard of the HPV vaccine, 27% had adolescent child(ren) who had received at least one dose, and 14% had child(ren) who had completed the HPV vaccine three-dose series.
• Most mothers were aware of the purpose for HPV vaccination (to prevent the HPV infection and cancer).
• Lack of information about the HPV vaccine guidelines (age, gender, number of doses, dosing intervals, etc.).
Varying levels of knowledge:
• Higher rates of ever hearing of the HPV vaccine among the insured, English speakers, and higher annual income.
• Higher rates of receiving the HPV vaccine among the insured and mothers who ever heard of the HPV vaccine.
Sources of information:
• The most common source is the child's doctor.
• Other sources: posters, brochures, internet, TV commercials, peers, and family members.
• The majority did not see HPV vaccine advertisements in Spanish magazines.
• Some concern that schools do not inform parents about the HPV vaccine.
Attitudes toward the HPV vaccine
• Overall high acceptability for the vaccine for both female and male adolescent children.
• Concern that the HPV vaccination age of 11 is too young; preferred to be given around the time of puberty.
• Agreement that the vaccine does not encourage sexual activity.
• Mothers are the primary and sole people making decisions about their children's vaccines.
Barriers and facilitators of HPV vaccination
Barriers
• Lack of recommendation from the HCP for the HPV vaccine.
• Lack of available information on the HPV vaccine in Spanish.
• Concern about potential adverse and vaccine safety.
Facilitators:
• HCP recommendation as the strongest facilitator.
• The mothers' desire to protect their children from the HPV infection and HPV-related cancers.
Strategies to improve HPV vaccination
• Improving HCP recommendation through providing information, addressing concerns about side effects, and presenting a strong recommendation and support for the HPV vaccine.
• Using print materials on the vaccine such as booklets and brochures.
• Community outreach activities to educate mothers about the HPV vaccine.
• School-based approaches to educate mothers about the HPV vaccine.
• Using mobile technology (e.g., apps and text messaging) to educate mothers about the HPV vaccine and remind them of upcoming appointments.
• Setting up follow-up visits immediately after receiving the first dose to improve HPV vaccine completion.

CL, Caribbean Latina; SCA, Central/South American; HCP, health care provider.

## Results

A total of 132 Latina mothers participated in 14 focus groups—9 groups of SCA mothers (58.3%) and 5 groups of CL mothers (41.7%). The participants' countries of birth included Columbia, Cuba, the Dominican Republic, Ecuador, El Salvador, Guatemala, Honduras, Mexico, Peru, Uruguay, the United States–Puerto Rico, and Venezuela. See Table 1 for additional demographic characteristics of the study participants.

### General knowledge about the HPV infection

The mothers in the study lacked knowledge about HPV infection, symptoms, and methods of transmission. Most of the women who indicated they had heard of the infection did not know what the acronym stood for, the mode of transmission, and ways in which to prevent it. Only a few mothers were aware of the consequences of the HPV infection, mainly related to cervical cancer. There was also some confusion between HPV and HIV. However, there were varying degrees of knowledge across Latina subgroups. For example, CL mothers appeared more knowledgeable about HPV infection than SCA mothers.

#### Confusion about transmission

*But I believe that we are all born with the human papilloma and some develop it and others do not*.–SCA mother

#### Confusion about symptoms

*When you have the infection sometimes it is present (obvious) and sometimes it is not present… I mean is that true? My friend said something like this but I'm not sure. I don't even know what symptoms. And when there are no symptoms, how do you know if you have it? I don't know exactly*.–SCA mother

#### Confusion between HPV and HIV

*I know that the contact of this HPV disease is not only by body to body but also by injecting a person that is infected and then vaccinating another person, also they can be infected*.–SCA mother

#### More knowledge among CL mothers

*There's different types of HPV… So your body, depending on the type, can fight that virus. For some women. But, if your body doesn't fight it, that's when it can cause cervical cancer*.–CL mother

In addition, there was great uncertainty among the mothers in the study related to their knowledge about the HPV infection. Some of the women were unsure of who would be at risk for the infection whereas others were unsure about the transmission. When asked whether the HPV infection affected women, men, or both genders, half of the mothers thought it only affected women whereas half thought it affected both genders.

#### Uncertainty about who is at risk

*I thought older women got it, some say that only recently in children and that I didn't know, only knew, thought that only women. I also heard that it was the man, that transmitted it to the woman, and that it was in the women that developed but I didn't think that it was the young, but the adults*.–SCA mother

#### Uncertainty about the transmission

*It's not only contacted by sexual intercourse there's a lot of other ways that you can contract it. Umm, I not really sure about that [what some of those ways are]… I don't really remember*.–CL mother

Mothers in the study lacked knowledge of how HPV is transmitted. Even though several women were aware that the infection is transmitted sexually, some were misinformed about transmission. Some mothers thought the HPV infection is transmitted by causal contact/touching, through childbirth, exchanging underwear, receiving a blood transfusion, sharing needles, and through use of public facilities. Further, SCA mothers were less knowledgeable and had more misinformation about the HPV infection.

*[Transmitted] By contact, with another person, that is the person that has it infects the other*.–SCA mother

*[Transmitted] by means of the hands*.–SCA mother

*Another way of transmitting that disease is by means of the mothers themselves, when they do not take preventive measures in time; they do not take care of themselves. Because of that, then obviously the children are born with the infection. I had a case of a grandchild. My, my daughter-in-law did not get treated in time and when the birth came and the little one was born with that physical problem. And then soon after, ah each one is in treatment*.–SCA mother

*It [HPV infection] is transmitted by needles*.–SCA mother

*It can be contracted by exchanging panties [several women agreed]. They also say there that the virus comes and if another person uses it they can catch the virus*.–SCA mother

*I knew that by a blood transfusion*.–SCA mother

*The bacteria can transmitted in public places such as swimming pools where we all go and get wet in the summer*.–CL mother

When asked to describe the consequences of the HPV infection, mothers who knew about the HPV infection also knew that HPV causes cancer. Although the cancer most often cited was cervical cancer, a few mothers mentioned that HPV leads to oropharyngeal cancer. Further, CL mothers appeared more knowledgeable about the consequences of the HPV infection.

*If it's not detected in time, it can cause cervical cancer, it can cause oral cancer…There's different strands of HPV. So your body, depending on the strand, can fight that virus…But, if you don't, if your body doesn't fight it, that's when it can cause cervical cancer.*–CL mother

*I believe Michael Douglas, who has cancer of the throat by the same papilloma virus*.–SCA mother [a few other women agreed]

In addition to correctly identifying that HPV could cause both cervical and oropharyngeal cancers, there were many mothers who believed that uterine and ovarian cancers were caused by the HPV infection as well. Further, a few mothers noted that HPV could also cause genital and oral warts.

*It shows up like some sort of sores, in the genitals of the person and that she needs to apply some medication for them to disappear, but that is to say, the virus is always there and they can come back at any time.*–SCA mother

Although most mothers had not heard of the HPV infection, those who had said that they learned about it through their child's health care provider (HCP). In addition to receiving knowledge about the HPV infection from their child's HCP, some women stated that they had heard about the infection in the media, specifically on the internet or on TV commercials (*It's now you hear it more. You hear it on TV, you hear commercials, you know it's more out there now.*–SCA mother). A few women said they knew others who were infected with HPV, which prompted them to seek out more information, either by speaking with their child's doctor or by looking it up on the internet.

*I read about it in a doctor's office. Yeah, in the doctor's office. A pediatrician told me himself and he suggested it. I never heard about it.*–CL mother

*The doctor when he brought it to my attention when [my son] turned 11 that's when I was like ok wait–alright, so I'm gonna get this because it's really going around and I knew somebody who had it.*–CL mother

### Knowledge about the HPV vaccine

At the beginning of the focus group discussions, participating mothers completed a demographic data sheet that asked whether they had ever heard of the HPV vaccine and whether their adolescent child(ren) had been vaccinated against HPV. Only 55% of the mothers indicated that they had ever heard of the HPV vaccine, 27% indicated that their adolescent child(ren) had received at least one dose of the HPV vaccine, and 14% indicated that their child(ren) completed the HPV vaccine three-dose series. Results of the bivariate analysis are illustrated in Table 3. Ever heard of the HPV vaccine was significantly higher among mothers who are English speakers, have higher income, and have health insurance. Receiving at least one dose of the HPV vaccine was significantly higher among those who were insured and who had heard of the HPV vaccine.

In the focus groups, most mothers stated that they had heard of the HPV vaccine and knew that its purpose was to prevent HPV infection and cancer. *It is against the papilloma virus. And it is a virus that has been detected recently, that produces cancer,* said an SCA mother. Although some mothers said that they had heard about the vaccine from the internet or television commercials, most mothers who knew of the vaccine heard about it from their child's HCP. Still others said that they first heard about the HPV vaccine from their peers and/or family members. One of the CL mothers explained:

*The first time I heard about this vaccine was from an aunt in Texas when my cousin was 12 or 13 and she was commenting that she was taking my cousin to the clinic to get her the papilloma injections because our family has a history of cancer, and it is after that that I started to inquire about the vaccine. In the end I found out that she had given it to her but I didn't pay too much attention because my son was too young then. It wasn't until my son reached 12 years old that the pediatrician examined him and said he needed it. So at the beginning, I heard it from my aunt*.

Some mothers expressed concern that the schools had not informed them of the HPV vaccine and its importance. They explained that although school nurses provide health information to parents about various health topics and particularly vaccinations, they have not had interactions with school nurses in which the HPV vaccine and its importance was mentioned or discussed. One of the SCA mothers explained:

I believe that many of the diseases from viruses, and of those things that happen to young people is due to a lack of information. And the prevention that they get they do not get it from school, and lamentably, this supposedly developed country, it is a power, but in the Latin American countries, the information is available in the schools and colleges. But here I haven't seen anything. My children are already grown but I have 3 grandchildren and I take care of them and the girl is 10 and has not received any information about that. I think that it is here in this country that the information is lacking, in our country they inform us from foot to head, they are informing us all the time, it is this country which doesn't inform about anything.

Mothers were asked about the HPV vaccination guidelines regarding age, gender, number of doses, and durations between doses. Although some mothers knew that the vaccine should start around age eleven, most thought the vaccine was only supposed to be administered to girls. Only a few mothers knew that the vaccine had three doses and none of the mothers knew the timeframe within which all three doses of the vaccine should be administered.

*What age? I think I heard it is at nine.*–CL mother

*Children should be vaccine from nine years on up.*–CL mother

*I thought that it was up to 22 years old or 24 that they could receive it because my daughter is 21 or 22 and, I have not gotten it for her thinking that that was the limit, it is news to me that it is up to 26.*–SCA mother

*Hmm, boys or girls? I think only the girls should get the vaccine.*–SCA mother

*I think it is three vaccines, every two or three months. The doctor picked the dates for us. I'm not sure exactly the time between the shots.*–SCA mother

*Quarterly? I think it's less than 6 months, I mean my daughters got it closer than that. I don't remember. I think maybe every 3.*–CL mother

The majority of mothers indicated that their child's physician is the main source of information about vaccines, including the HPV vaccine. A few mothers said that they learned about the HPV vaccine from posters and brochures they saw at doctors' offices or health centers. Several mothers indicated that they learned about the HPV vaccine from TV commercials. The majority of mothers indicated that they did not see HPV vaccine advertisements in Spanish magazines.

*The doctor told us about this vaccine. She told us it was important before they became sexually active, that it helped to prevent infection. I get most of my information about vaccines from the doctor.*–SCA mother

*I would talk directly with my daughter's doctor, not to a friend or someone else. I only trust what the doctor says… I would make this decision talking to the doctor.*–SCA mother

*Well I heard this in the school. They formed a group in the school for mothers with a doctor when my son was in the sixth or seventh grade. It was for mothers.*–SCA mothers

*I heard about it on TV. It was an advertisement on one of the channels in Spanish.*–CL mother

*I have heard about it on the TV commercials, and from mothers of some of my daughter's classmates.*–CL mother

### Attitudes toward HPV vaccination

Overall, all of the mothers were receptive and believed that the vaccine would be beneficial and should be given to adolescents. Most mothers thought that the vaccine should be given to both males and females. Some of the mothers believed that the vaccine should be given around the time of puberty. Specifically, in the case of females, mothers agreed that adolescent girls should receive the HPV vaccine as soon as they began to menstruate. All mothers agreed that giving the adolescents the vaccine was not giving them permission to have sexual intercourse. Two SCA mothers explained:

Because that is only a protection, that doesn't mean, doesn't mean that you are giving them an open door to go do it.

I feel more secure with my daughter, it is not that I tell her the idea that some people are talking about, and that she should go and have sexual relations. This is to prevent something in 5–10 years from now when my daughter gets married and she has to be cautious that her husband, whoever he may be, is not going to transmit this infection to her. At least she will be protected, and maybe after ten years I will tell her because right now she has no idea what this vaccine is for because I just told her that it was to prevent infections like the vaccine for the measles or the flu. I cannot tell her: my daughter it is for you to go and have sex! I say that it is ignorance on the part of those who tell me that I am encouraging her.

In fact, some of the mothers expressed that because children were having sex at a younger age, they needed all available protection. They felt that the HPV vaccine would be necessary to protect kids in case they become sexually active without their parents' knowledge. One of the CL mothers explained:

But from what I am hearing I suppose that the parents have to be intelligent and intuitive and they should give it to them before the children start to have sexual relations. The kids in this country start having sex early and it should be given in early adolescence… I think pre-teens. Like twelve. You know what? I'm, I'm going to say either earlier, ‘cause I came home one day and I found… a used condom in my garbage. [My son said] mom it's mine. And it was devastating, devastating to me. Because he was twelve years old.

Several mothers were concerned about the young age at which the HPV vaccine was administered. They felt that 11 years of age was too young for the vaccine and stated they would be more comfortable giving the HPV vaccine to their children at a later age. One SCA mother said, *I say from fourteen on is all right.* Another SCA mothers stated, *I would say the girls after puberty, after they become young misses [most mothers agreed)… I believe the same for the boys—between 16 and 18.*

Mothers in the focus groups were asked about who is usually involved in the decision-making process for their children's vaccination. Most mothers indicated that they are the primary and/or sole person making these decisions. A few mothers indicated that although they might consult with their children and husbands before vaccination, it is ultimately the mother's decision. They further explained that even if the child or father disagreed with the decision to vaccinate, they would vaccinate their children if they thought it would be beneficial. One of the SCA mothers explained:

I would talk with them, with my daughter and my husband, that is giving them the information that I am going to get today, giving the information to my husband, explain to him why it is necessary what the girl needs to get the vaccine, and to the girl also, because she has begun to ask questions, mother why does the doctor pinch us or why this, then I would have to also explain the reason why OK. Well with the children's father, who is not my husband, I have another partner and I also tell the children, I don't ask them for permission to give it to them but I tell them why we are going to give it to them because 11 and 12 year old children are for example already different, different times that they are giving it to them, at least to explain even though it may be over their heads, why we are going to give you the vaccine and taking care of what.

Mothers were asked about other sources of influence on their decision to vaccinate their children, such as religion, friends, and the media. Most mothers indicated that religion usually does not influence their decision whether to vaccinate their child. However, they did state that friends who have had negative experiences with the HPV vaccine would have some influence over their decision. An SCA mother explained:

I have many opinions from other friends who tell me that they have had reactions or, they don't want to give it to them because inclusively I have a friend that her doctor told her not to give it, why would her own doctor say no? And then, I remained with, that doubt… oh, no? That is why I have not given it to the other [daughter].

### Barriers and facilitators of HPV vaccination

Lack of information was cited as the most common barrier to HPV vaccination. Most mothers indicated that although they were aware of the HPV vaccine, they needed more information, specifically information in their native language, before vaccinating their children. This also included concern about potential adverse and vaccine safety, mainly due to lack of information on these concerns.

*I believe that many of the diseases from viruses, and of those things that happen to young people is due to a lack of information. And the prevention that they get they do not get it from school, and lamentably, this supposedly developed country, it is a power, but in the Latin American countries, the information is available in the schools and colleges. But here I haven't seen anything. My children are already grown but I have 3 grandchildren and I take care of them and the girl is 10 and has not received any information about that. I think that it is here in this country that the information is lacking, in our country thy inform us about from foot to head, they are informing us all the time, it is this country which doesn't inform about anything.*–SCA mother

*I got it one time only as I was telling my friend, my son fainted and had a little reaction to the vaccine, he fainted, he got some type of attack and I did not go back for the other two doses that followed. That is why am concerned to learn what is going on, what's happening?*–SCA mother

*The doctor told me to give her the vaccine, I asked my husband and he said get it and the moment that she got it she fainted…When the nurse got scared she called the doctor, they awaked her but before she came to she had some kind of attacks and she woke up crying, with a panic and I felt responsible that she could have gotten something more severe.–*SCA mother

Mothers indicated that another major barrier for HPV vaccination was lack of HCP recommendation. They explained that in most cases their children's HCPs either did not mention the HPV vaccine or presented it as an optional vaccine, providing very little information. A few mothers stated that their children's HCP had negative views about the HPV vaccine, which deterred them from considering the vaccine for their children.

*The pediatrician said that if I didn't want it, I didn't have to get it. But since the pediatrician has been with us since my children were born. [I asked] What do you recommend? I asked because he speaks Spanish and then he says no.*–SCA mother

In addition to identifying barriers to HPV vaccination, mothers were asked about facilitators for HPV vaccination. Most mothers identified HCP recommendation as the strongest facilitator. They explained that the opinion of HCPs and the way they present the HPV vaccine creates great bearing on their decision-making process.

*I ask the doctor. If he tells me that she needs it and tells me all the reasons why and I ask him all the questions I can think of and I see that it is for her own good, then I say go ahead and give it to her. I like to know what I am exposing my daughter to, and once I know it is to protect her from something then I tell him that I agree and to give it to her.*–CL mother

Another facilitator for HPV vaccination was mothers' desire to protect their children from the HPV infection and HPV-related cancers. Most mothers expressed their desire to take on any action to protect their children's health and well-being in the short- and long-term future. One of the SCA mothers explained:

*This is to prevent something in 5–10 years from now, when my daughter gets married and she has to be cautious that her husband, whoever he may be, is not going to transmit this infection to her. At least she will be protected, and maybe after ten years, I will tell her because right now she has no idea what this vaccine is for because I just told her that it was to prevent infections like the vaccine for the measles or the flu.*–SCA mother

### Strategies to improve HPV vaccination

The mothers in the study were asked what they thought would be effective methods to improve HPV vaccination among Latino adolescent children. Most mothers indicated that HCP recommendation for the vaccine was the key factor in their decision-making process and would consequently be the most influential way to improve HPV vaccination. To that end, the mothers deemed it necessary for HCPs to provide information about the vaccine and address parental concerns about potential side effects. They also wanted to hear that HCPs recommend and support HPV vaccination. In addition, several mothers thought that HCP recommendation could be supplemented with print materials about the vaccine, such as booklets and brochures.

*I wouldn't know what Human Papilloma is. What is it? That should be included [in talking to the doctor]. What it is and what are the, the risks and you know, Jennifer [pseudonym for another participant] saying that there's so many strains of it. What are the strains? What can these strains cause in your body, like, we all know maybe it causes cervical cancer but what else? If there's so many strains it's not just cervical cancer. And, and going through the whole routine thing… Me personally, I like to be informed when I go to something, not just, this is what you got to do*.–CL mother

*If I have to do this, why do I have to do it? What are the benefits, what are the risks? Like, definitely doing some kind of brochure that shows like, everything, and going through it with the community when you educate.*–CL mother

Several mothers indicated that community outreach activities would be helpful in improving HPV vaccination, such as talking to mothers and distributing brochures about the HPV vaccine at community centers or health fairs. Several women also suggested that a school-based approach to educating mothers about the vaccine would be widely effective in increasing HPV vaccine knowledge and awareness among a large number of parents and adolescents. This could be done in collaboration with school nurses or parent–teacher associations or groups.

*I think this should be included in the sex-education. They talk about when your menstrual comes, and other things, you know. They should start talking about this vaccine too.*–CL mother

*The [school] nurses should do that but in the school that my son was at, the nurses only spoke English… The nurses in the school are the ones in charge and they should also be giving out information.–*SCA mother

*I think that it is the responsibility of the school to notify the parents that their children are approaching the age where they need to get this vaccine. They should have a program to teach the parents to be involved in the lives of their children. In this case, to inquire of the parents if they know about this vaccine, and if they don't, they should invite them to a meeting. The parent who considers himself responsible for his children will attend that meeting to learn what this is all about.*–SCA mother

Because smart phones were widely used among mothers in the study, most mothers expressed interest in using mobile technology, such as apps and text messaging, to learn about HPV vaccination. In addition to using mobile technology to raise awareness about HPV vaccination, most mothers thought it would be beneficial to have mobile technology set reminders for follow-up appointments to improve completion of the multi-dose series of the vaccine. Lastly, several women suggested that would be helpful to set up follow-up visits (i.e., appointments for the second and third doses) immediately after receiving the first dose to improve HPV vaccine completion.

*It would be helpful to have a text message come, if you have a smart phone with like a link where you can open and read about the vaccine.*–SCA mother

*There are times with the messages on cell phones when they send you a reminder. There is a way that you press that same message and it gives you the information about the vaccine right there and then.–*SCA mother

*The way I completed all three doses of the vaccine was because I was called from the hospital as a reminder to come back and complete the vaccine.*–CL mother

## Discussion and implications

This study provides rich qualitative findings on HPV vaccination knowledge and attitudes from low-income Latina mothers from varying Latin American/Caribbean regions. Results show that overall knowledge about HPV infection and vaccination was low among Latina mothers. This is consistent with prior studies suggesting low levels of knowledge about HPV vaccination among Latino parents^21,23,26,40^ This was also evident in other qualitative research that reported low levels of knowledge around HPV infection and vaccination.^29,30^ In addition to lack of knowledge, the findings indicate various misinformation about the transmission of HPV infection, such as through childbirth and the use of public facilities. Moreover, this study provides important new information about variations in HPV vaccination knowledge across Latina subgroups. In particular, SCA mothers were less knowledgeable about the HPV infection and its consequences as well as the HPV vaccine, and had more misinformation about the HPV infection and vaccination, compared with CL mothers. Further, similar to the findings of other studies,^23,40^ we observed high HPV vaccine acceptability among low-income, Latina mothers. Nevertheless, several mothers in our study expressed the need for more information, such as potential side effects and vaccine safety.

More importantly, Latina mothers in our study indicated that HCP recommendation is an important factor to getting the HPV vaccine for their children. These findings demonstrate the prominent role that HCPs play is affecting HPV vaccination, through which HCP recommendation is considered the main facilitator; and the lack of HCP recommendation is considered the main barrier for HPV vaccination. This is consistent with other studies^24,25,41,42^ in that HCP's recommendation is the key determining factor in the mother's decision to vaccinate her children. This is also consistent with the findings of other qualitative research, in which Latina mothers reported lack of provider recommendation as the main barrier to HPV vaccination.^30^ Further, other qualitative research has shown that the most commonly reported barrier among Spanish-speaking parents is when providers do not encourage the initiation of the HPV vaccine or do not explain the necessity of completing the series.^31^ In addition, the mothers in our study identified their desire to protect their children, which ties to the perceived severity of HPV, as a facilitator to HPV vaccination, which is consistent with other studies.^25,43^

Although national data suggest that Latino populations have higher rates of initiation, research in the Greater Newark area revealed that only 21% of Latino adolescents (10–20 years old) have initiated the HPV vaccine,^18^ and 8% have completed the three-dose series.^19^ Further, our study findings indicate that subgroup variation in knowledge and attitudes within the Latino population may lead to heterogeneous vaccine uptake. To improve vaccine initiation rates among low-income Latina children, culturally appropriate intervention strategies should focus not only on educating all Latinas from varying regions but also on improving provider communication with mothers about HPV infection and vaccination. Our discussions with Latina mothers about acceptable strategies for improving uptake also provide actionable information for increasing uptake in this community. The use of smartphone technology, such as mobile apps and text messaging to educate mothers and send vaccination reminders, is welcomed by the Latina mothers in this study. Several studies^44–46^ have implemented and evaluated text message and reminder systems and have found them to be an effective way to improve HPV vaccination coverage. However, such interventions may be costly and may require higher levels of literacy, particularly among populations with lower education levels and more recent immigration to the United States. Alternatively, community and school-based outreach strategies may be more beneficial, cost-effective, and easily achievable. Lastly, as is evident from the results of this study, providers play a crucial role in the vaccination decision-making process. Therefore, interventions targeting HCPs on HPV vaccination^47–49^ present a critical approach to combat disparities in HPV, thereby decreasing the prevalence of HPV-related cancers in the Latina population.

Our study is one of the few that has focused on understanding HPV vaccination knowledge and attitudes among low-income Latina mothers from varying Latin American/Caribbean regions. However, some limitations of the study should be noted. First, we did not collect data on HPV vaccine uptake; therefore, it is unclear whether mothers' reported barriers and facilitators to vaccination matched their actual past and/or future HPV vaccination behaviors for their children. Second, the CDC guidelines were updated from a three-dose to a two-dose completion regimen between the initiation of this study and the completion of data analysis. There is a possibility that this change may affect parental attitudes toward the vaccine. However, knowledge about HPV infection and attitudes toward strategies to reduce barriers to vaccination are less likely to be impacted by the guideline changes. Finally, our study focused on South/Central American and CL mothers recruited from a large community health clinic and an urban, hospital-based clinic. This population may not be generalizable to Latina mothers of different descents and/or in other settings. Despite these limitations, few studies to date have focused on the heterogeneity of Latin American subgroups and the potential variation that may exist in HPV vaccination knowledge and attitudes across subgroups. Our large low-income sample of diverse Latina mothers provides valuable insight to the need for additional research on the heterogeneous Latino population and HPV vaccine uptake.

In conclusion, the findings of our study support the need for multicomponent interventions to address low HPV vaccination in all populations, but particularly for low-income, racial/ethnic minority subgroups with compounding social and demographic barriers to health information and HPV vaccination. Our findings suggest that improved provider communication with Latina mothers about HPV infection and HPV vaccination combined with strategies to motivate and remind Latina mothers to vaccinate their children are not only needed but also suggested directly by mothers from these communities. Multi-dimensional HPV vaccine interventions to date have primarily focused on increasing mothers' knowledge about the HPV vaccine, assistance with navigating the health care system to access the HPV vaccine (e.g., nurses, program coordinators), educating providers about the HPV vaccine and HPV/cervical cancer epidemiology, and using quality improvement techniques to improve provider–parent communication.^50,51^ However, more research is needed to evaluate whether these strategies can be adapted to effectively improve HPV knowledge and vaccination among Latina mothers of different descents in low-income, urban areas of the Eastern United States. Additional efforts are needed to adapt and accelerate the implementation of evidence-based HPV vaccine promotion strategies in varying settings and for vulnerable subgroups, including diverse Latina mothers in community health settings that serve low-income populations in urban areas.

## Abbreviations Used

CL: Caribbean Latina
HCP: Health care provider
HPV: human papillomavirus
SCA: South and Central American
